# Supplementary material for: Comparative analysis of oral health behaviour and utilisation of oral health care services in the general population and among patients with non-communicable diseases in Korea: a repeated cross-sectional survey conducted from 2008 to 2022
Source: PLoS One. 2025 Apr 22;20(4):e0321816. doi: 10.1371/journal.pone.0321816 (PMC12013916; doi:10.1371/journal.pone.0321816)
Supplement: Attachment — Submitted filename: Review comments response_20241223.docx [file pone.0321816.s007.docx]

Review Comments to the Author

Please use the space provided to explain your answers to the questions above. You may also include additional comments for the author, including concerns about dual publication, research ethics, or publication ethics. (Please upload your review as an attachment if it exceeds 20,000 characters)

**Response: We would like to express our sincere gratitude for your valuable feedback and constructive comments on our manuscript. We have revised our manuscript in accordance with reviewers’ suggestions.**

Reviewer #1: The study provides a crucial and timely examination of oral health behavior and service utilization among patients with non-communicable diseases (NCDs). Given the global rise in NCDs, this research holds significant relevance to both clinical practice and public health, highlighting the disparities in oral health care that may exist for this vulnerable group. The analysis is bolstered by the use of a robust dataset from the Korea Community Health Survey spanning a substantial 14-year period (2008–2022), offering a comprehensive view of trends over time. This extended period enables meaningful insights into how oral health behaviors and utilization of services have evolved, both in the general population and in patients with NCDs.

One of the study's major strengths is its identification of the disparities in oral health behaviors between the general population and those with NCDs. By demonstrating that NCD patients generally exhibit poorer oral health behaviors, the research points to specific areas for intervention, which is essential for developing tailored strategies to improve their oral health outcomes. Furthermore, the study has significant public health implications, as it provides an evidence-based foundation for healthcare policymakers. Identifying gaps in oral health services for NCD patients paves the way for strategies that can enhance preventive care and improve access to services, ultimately leading to better overall health outcomes for these patients.

**Response: Thank you for your valuable feedback. As mentioned, we expect that this manuscript will contribute to establishing strategies to improve oral health in NCD patients. The manuscript has been revised according to your comments below.**

However, some critical points require further attention to maximize the study's impact:

Lack of Specificity in Patient Subgroups: While the analysis covers 15 different NCDs, the abstract does not clarify whether specific trends or significant differences were observed among the various NCD subgroups. It would strengthen the study to explore whether particular conditions, such as diabetes mellitus or hypertension, have distinct oral health behaviors or service utilization patterns. Additionally, these 15 NCDs could be grouped into major categories (such as metabolic, cardiovascular, and mental health conditions) for more granular analysis. Since oral diseases share common risk factors with several NCDs, a subgroup analysis could reveal important clusters of risk factors and provide insights into how interventions might be more effectively targeted.

**Response: Thank you for your valuable suggestion. As you mentioned, it is important to examine which types of NCD patients have more problems with oral health behaviour. Therefore, we focused more on the oral health behaviour of patients with hypertension or diabetes, which are the most common types of NCD patients (Page 14~). However, we acknowledge that the current Discussion section is categorized based on individual oral health behaviour rather than disease-centered. Accordingly, we added more content to Discussion section to monitor the oral health behaviour of more vulnerable disease groups among various types of NCD patients (Page 21).**

**Furthermore, it would be meaningful to divide the types of NCD patients and check whether there are differences in oral health behaviour between subgroups. However, the primary purpose of this study was to focus on comparing and analyzing trends in health behaviour in various types of NCD patients. Therefore, we did not perform statistical analyses such as cluster analysis to divide the types of NCD patients separately. However, we believe that your suggestions are worth considering in future studies. We have added suggestions for subgroup analysis in Discussion section (Page 24).**

Generalized Recommendations: The abstract concludes with a recommendation to implement educational and promotional measures, but these suggestions are somewhat broad. Providing more specific or innovative strategies tailored to the needs of NCD patients could enhance the recommendations' impact. For instance, highlighting which specific oral health behaviors or service utilization gaps—such as annual scaling or regular oral examinations—should be prioritized for intervention would make the recommendations more actionable.

**Response: As you suggested, we have made our suggestions in Conclusion section of the Abstract more specific (Page 5).**

Comparison with General Population: Although the comparison between NCD patients and the general population is briefly mentioned, the abstract does not delve deeply into the implications of these differences. A more thorough discussion of why oral health service utilization rates are lower in NCD patients, whether due to access issues, awareness gaps, or other barriers, could provide valuable insights that inform future public health strategies.

**Response: The abstract, which has a word count limit, could not fully cover the results. We checked whether the results section of the abstract was more specific and revised the description accordingly (Page 4).**

Limited Focus on Policy Implications: While the study emphasizes the need for improved oral health behaviors in NCD patients, it could better emphasize the potential policy changes or adjustments in healthcare systems necessary to address these gaps. Given the increasing global burden of NCDs, incorporating a discussion on the importance of medical-dental integration and coordinated care for patients with NCDs would significantly strengthen the study. Policy suggestions that focus on better integrating dental care within overall NCD management could offer long-term solutions for improving health outcomes in this population.

**Response: We also strongly agree with the importance of medical-dental integration and coordinated care for patients with NCDs that you mentioned. That is why we have already emphasized the importance of oral health care services in in- and outpatient management programmes for patients with NCDs (Page 22). Here, we have put more emphasis on the integrated provision of oral health care services.**

The following suggestions may further enhance the clarity and impact of the study:

Provide more specificity on differences in oral health behavior and service utilization across distinct NCD subgroups to offer more targeted insights. (to explore whether Major NCDs and other NCDs can be subgroup in the analysis)

Strengthen the policy implications by discussing potential strategies for medical-dental integration and more coordinated care for NCD patients.

Clarify and expand upon the regional variations identified, including potential reasons for these disparities and tailored approaches to address them.

These revisions will further solidify the paper’s contribution to the field and its practical relevance for addressing oral health gaps in NCD care.

**Response: Thank you again for your progressive suggestions that can enhance the policy application of the research. In addition to the subgroup analysis of NCDs that you suggested earlier, we believe that medical-dental integration and more coordinated care for NCD patients are review comments that can develop the results of this study into actual policies. We reviewed and revised the related descriptions in Discussion section to include specific policy suggestions (Page 22).**

**Furthermore, we agree with the opinion that we should pay more attention to the differences in oral health behaviour among NCD patients between regions confirmed in this study. Therefore, we separately described the differences in oral health behaviour among provinces and cities in Korea in Results section, and emphasized the need to prepare measures for vulnerable areas of oral health behaviour and oral health care services in Discussion section (Page 22). We reexamined the description of these contents and refined them to deliver a clearer message (Page 23)**

Reviewer #2: 1. The authors have used available survey data from community health surveys and tried to capture the trends/changes in oral health behaviour among those with non communicable diseases (NCDs) and among the other general population. A problem with public health surveys as compared to registry-like data is that the sample is bound to change every year, (900 who are sampled per public health center, will change every year!). Within the limits of the available data, the authors have tried to capture the trends using a suitable statistical trend analysis/regression.

**Response: Thank you for your valuable feedback. As you pointed out, the Korea Community Health Survey does not use a panel survey method but rather a repetitive cross-sectional survey method. We have already mentioned in Discussion section that this survey method has a limitation in that it is difficult to identify the causal relationship between disease diagnosis and oral health behavior (Page 23). However, the large sample size of the Korea Community Health Survey can be seen as increasing the validity and generalizability of the analysis results. This methodological advantage has also been mentioned in Discussion section (Page 18).**

2. The main issue in the analysis that I noticed is that the change in oral health behaviours is on the up-trend for all population but, those with NCDs it is still less. In addition to the "self reported" nature and the associated false reporting by people, the other thing is that those with highest risk (among the NCD and non NCD population) may not be captured because they are either not coming into the system or they are lost... Also, this self-reporting will also under-report those who have not yet been identified or diagnosed with NCD, which in high probabiility are people who do not access the health system. These limitations should reflect in the discussion.

**Response: The Korea Community Health Survey relies on self-reports to collect responses related to health behaviors. This limitation may result in low validity of the responses. We have already addressed this as a possibility of social desirability bias in Discussion section (Page 23). However, as you mentioned, self-reports may also affect the determination of whether or not a person has a disease. In other words, we need to be careful when estimating the prevalence of NCD patients. We have added this limitation to Discussion section (Page 23).**

3. Some NCDs have a higher morbidity and higher association with poor oral health. In addition, NCDs being a lifestyle related disease, especially hypertension, diabetes (DM); those with common risk factors may also have a poor oral health behaviour and this may reflect on their overall low use of health resources and their consequent poor oral health. The change in behaviour would not happen without targetted interventions. And hence, they will remain low. Some segments of population with mental health challenges, may also resort to poor self-care and ignore their usual care routines, including tooth brushing etc., The authors could explain such associations in the discussion. They have explained the possible causes for poor oral health habits among those with stroke, but different NCDs may affect their ability to maintain or access the system in different ways. For example, arthritis may affect their dexterity to brush well and also their mobility and hence decreased access. Those with another NCDs may have different challenge, and is not discussed even in broad sense. A table to highlight this or a short paragraph on the same will be good, and this should be loco-regionally informed by prior data or known experience of the authors and not just speculation.

**Response: In this study, we examined the trends in oral health behaviours and oral health care service utilisation for patients with various types of NCDs. We also summarized the implications for each oral health behavior and oral health care service utilisation. Although we presented the results focusing on diabetes and hypertension, which have large patient volumes among NCDs, we acknowledge that we were unable to examine the characteristics of each disease in detail. Accordingly, we added more content to Discussion section to monitor the oral health behaviour of more vulnerable disease groups among various types of NCDs patients (Page 21).**

4. I feel, the policy level suggestions and system level changes suggested by the authors based on their work appear too generic and not data driven- as per the data presented. Also, with so much missing data in so many years and with information only on 'tooth brushing habits', it is difficult to justify major policy level recommendations. The strongest point is the recommendation that oral health has to be integrated with NCD screening. Majority of individuals in general population and those with NCDs have shown similar habits except some specific conditions such as stroke. The authors have themselves identified potential issues in this group of population. They have found a reverse trend in patients with arthritis & dyspidemia. The potential reason is that arthritis & dyslipidemia have both increased in prevalence since 2008, and a large number of individuals with good oral health habits may also have developed dyslipidemia and are now here, where-in their habits has not changed, just their categorisation. With respect to arthritis, the reason for increased in oral examination in previous years may be due to the pre-joint replacement oral health screening, to remove foci of infections prior to reconstruction/replacement (or possibly some other reasons).

**Response: We have presented the policy application of this study in a separate part called Policy Suggestions in Discussion section. As you suggested, there is a way to simultaneously perform NCDs screening and oral examination, but this study focused on the value of hospitalization and outpatient visits due to treatment of NCDs patients (Page 22). In a previous study, this was called a ‘wake-up call’ or ‘teachable moment’ and suggested as a good opportunity to improve the patients’ health behaviours. Therefore, we mentioned that the importance of oral health behaviours and the necessity of using oral health care services should be sufficiently addressed in the education of NCD patients in the inpatient and outpatient settings. However, we have revised and strengthened the policy suggestions considering your suggestion (Page 22).**

The authors have included 15 NCDs and have tried to explain a lot of changes, but during the discussion and conclusion they resort to generic suggestions than to base it on the data along with known trends in NCDs in their country. A suggestion would be to correlate the oral health behaviours to changing prevalence in NCDs which may reveal whether those with NCDs are actually neglecting their oral health. It is also prudent to highlight those conditions where the impact of poor oral health can be more detrimental. Atopic dermatitis, allergic rhinitis conditions with a poor oral health behaviour is a problem, but not so much as a person with DM or hypertension or stroke, where the risks may be higher.

The overall impact of the paper and the policy level suggestions they can provide can be better improved if the data is interprepted or discussed based on prevalence and change in prevalence of the NCDs themselves especially with respect to those with higher morbidity and stronger established association with poor oral health such as DM.

**Response: As you mentioned, changes in oral health behaviours and oral health care service utilisation should be considered together with changes in the prevalence of NCDs. However, as mentioned earlier, the Korea Community Health Survey, the data source for this study, uses a repeated cross-sectional survey method, which makes it difficult to identify the chronological relationship between the time of disease diagnosis and changes in oral health behaviours and oral health care service utilisation. This has been mentioned as a limitation of the study, and verification through panel surveys or registration data analysis has been suggested in follow-up studies in Discussion section (Page 23). Futhermore, as mentioned in the response to comment 3 above, the importance of monitoring vulnerable groups that need more oral health behaviours has been added to Discussion section (Page 21).**

5. What regional factors/system level factors affect their poor utilisation? Is there poor access? Or some other reasons, this is best explained by authors since they know the system better at their country.

**Response: This study reported variations in oral health behaviours and oral health care service utilisation among 17 regions in Korea. This study paid more attention to differences in results between urban and rural areas, and suggested strengthening oral care in rural areas with limited resources in the study area. This has been described in Discussion section, but the relevant content has been supplemented (Page 23).**
