# Supplementary material for: Comparative analysis of oral health behaviour and utilisation of oral health care services in the general population and among patients with non-communicable diseases in Korea: a repeated cross-sectional survey conducted from 2008 to 2022
Source: PLoS One. 2025 Apr 22;20(4):e0321816. doi: 10.1371/journal.pone.0321816 (PMC12013916; doi:10.1371/journal.pone.0321816)
Supplement: Attachment — Submitted filename: Review comments response_20250218.docx [file pone.0321816.s008.docx]

Reviewer #1: the comments to reviewer is satisfactory and the article presents important findings for medical dental integration.

**Response: We sincerely appreciate your time and effort in reviewing our manuscript. Thank you once again for your careful evaluation and valuable input.**

Reviewer #2: The authors have addressed the major concerns adequately. A few additional points for revision are submitted for the consideration of the authors.

**Response: We are grateful for your constructive feedback and suggestions. The manuscript has been revised according to your comments below.**

1. Abstract - Result section can be improved and may consider the following suggestions.

First part of the result section is on oral health behaviour. So, logically, the second can start with "Regarding Oral health service utilisation..." and follow it up with key results. The third part can be on "inter-regional" variations.

**Response: As you suggested, we have revised the sentences in the Abstract (page 4).**

2. Results pg 11 Line 4: From the study design, the total number is NOT 3,428, 968 patients, but datapoints (since around 200,000 per year are surveyed, with substantial overlap every year, which would be difficult to track). Unless, the survey method avoids resampling the same individual. And may consider either rephrasing the line 4 for total number of patients or may remove that from results to avoid confusion. Or may report the number of individuals sampled in the last survey alone. Or can mention, number surveyed in 2008 and the number surveyed in 2022.

Similar issues with the numbers presented for HTN, DM, arthritis etc., I think, the authors can use the LATEST survey result to summarise this data for the Table 1 and for reporting purposes. This gives the current or recent information that may be useful for policy decisions. The number of individuals with specific conditions in 2008 is less relevant compared to the number of people with the condition/disease in 2022. This revision will make the numbers easier to interpret. Table 1 may be updated in similar manner with latest data only, I feel.

**Response: We agree with the problem with the presentation method of Table 1. Instead of the existing method of presenting the number of patients by age group by disease, we have revised Table 1 to present the number of patients by year by disease. Accordingly, we have revised the sentences in the Results to emphasize the number of patients in the most recent year, as you suggested (page 11-15).**

4. Oral Health Behavior results: pg 14-15

The trends can be explained as a summary and since figures are self-explanatory, the important data alone may be highlighted and rest mentioned or labeled in figures. The information that is removed during this editing may be added to a supplement file under same headings.

Oral Health service utilisation and Inter-regional indicators may remain with sufficient detail as it is in the main article.

**Response: Taking your feedback into account, we have revised the sentence to make it more concise by removing duplicate numbers (page 16).**

5. Discussion: pg 18 Line 3: Main findings & significance of the study - The heading is redundant.

**Response: As suggested, we have removed that subheadings (page 20).**

6. pg 18 Line 4-6: The first sentence of discussion shall be the result and not what the authors did and may be removed or moved to next paragraph. The discussion may begin with line 7 in this page where the authors are summarising the study findings.

**Response: We have removed the first sentence in the Discussion section based on your review comment (page 20).**

7. The limitations section may be brought up before "interpretation and implications" so the reader may understand the limitations before reading the interpretations, and then policy suggestions followed by conclusion. Necessary minor changes may be needed to maintain the logical flow of the article/writing.

**Response: Thank you for your feedback. Depending on your preference, we would be happy to present the “Study limitations and scope for future research” before the “Interpretation and implications of principal findings.” However, it is customary to present the study limitations and suggestions for future research at the end of the Discussion section. We kindly ask for your understanding.**

8. pg 19-21 Implications This section has repeated much of the "Result section" information including the numbers. This repetition is redundant. This section can remove most of the statistical data, except the ones that are pivotal for further "policy recommendations" or "informing future research". Further sections on implications may also follow similar strategies of summary and implication instead of repeating the data from results section again.

Very important, very large or unexpected results/data may be retained for contextual purposes such as three fold increase or alarming decrease to emphasize or compare some key data. Otherwise, repetition of results data is redundant in discussion. In most paragraphs under "Implications", the second half of each paragraph contains information relevant to discussion section and first half has repetition from Results section. These lines may be removed for brevity and to remove redundancy.

**Response: We sincerely thank you for your valuable feedback. In response, we have reviewed and revised the sentences in the “Interpretation and implications of principal findings” section of the Discussion. Specifically, we have made the content more concise by removing unnecessary repetitions of the results (page 21).**

9. Pg 21; lines 6-9, 19,20 May be shifted to policy suggestion section and may add about "improving access".

**Response: In response to your feedback, we have relocated this content to “Policy suggestions” section (page 23-24).**

10. pg 21-23 Policy Suggestions

The policy recommendation section may be shifted to just before conclusion. Vulnerable and marginalized population specific recommendations may mention about improving access and promoting preventive oral care.

**Response: As mentioned earlier, we kindly ask for your understanding that the “Study limitations and scope for future research” is placed at the end of the Discussion section. We appreciate your understanding once again, as we have addressed both the limitations of the study and suggestions for future research simultaneously.**

11. The third limitation from pg 24 can inform policy recommendation about integrating medical-dental care and about need to include dental/oral health related items in the KCHS surveys. Bridging this data-gap is an important policy recommendation, I feel.

**Response: Thank you for your valuable suggestion. We have already emphasized medical-dental integration and oral medical care coordination in the first revision. As per your recommendation, we agree that it is crucial to conduct regular oral health-related surveys to monitor the oral health services that NCD patients are receiving, or not receiving. To reflect this, we have added a sentence highlighting the importance of monitoring the provision rate of oral health-related services (page 25).**

12. pg 25 Lines 1-13 Conclusion: I feel that the oral Health behaviour and utilising oral health services alone are not sufficient to prevent worsening of NCDs and I feel that the data in the study doesn't support the first line of conclusion. The authors may instead add a note on "common risk factors" as a link between oral health and general health. May take this suggestion, if agreeable, since this may appeal to a larger audience and it will be an effective way to push for integration of dental and medical care in the health system. Following this, the authors can also add a note on "integration of medical-dental health" in conclusion. In addition, the repetitive sentences from other sections of discussion may be removed or summarised and rephrased briefly. Lines 8-13 appear good and may be retained.

**Response: We sincerely appreciate your feedback. Taking it into account, we have revised the sentences in the Conclusion section (page 26). In particular, we have placed greater emphasis on the importance of medical-dental integration and oral medical care coordination for patients with NCDs.**
